# Supplementary material for: Manufacturing of high strength and high conductivity copper with laser powder bed fusion
Source: Nat Commun. 2024 Feb 12;15:1283. doi: 10.1038/s41467-024-45732-y (PMC10861549; doi:10.1038/s41467-024-45732-y)
Supplement: Supplementary file 1 — Supplementary information [file 41467_2024_45732_MOESM1_ESM.docx]

**Supplementary Information**

**Manufacturing of High Strength and High Conductivity Copper with Laser Powder Bed Fusion**

Yingang Liu^1,†^, Jingqi Zhang^1,†^, Ranming Niu^2,3*^, Mohamad Bayat^4^, Ying Zhou^5^, Yu Yin^1^, Qiyang Tan^1^, Shiyang Liu^1^, Jesper Henri Hattel^4^, Miaoquan Li^6^, Xiaoxu Huang^7,8^, Julie Cairney^2,3^, Yi-Sheng Chen^2,3^, Mark Easton^9^, Christopher Hutchinson^10*^, Ming-Xing Zhang^1*^

^1^School of Mechanical and Mining Engineering, The University of Queensland, St. Lucia, Queensland, Australia.

^2^Australian Centre for Microscopy and Microanalysis, The University of Sydney, Sydney, Australia.

^3^School of Aerospace, Mechanical and Mechatronic Engineering, The University of Sydney, Sydney, Australia.

^4^Department of Mechanical Engineering, Technical University of Denmark, Lyngby, Denmark.

^5^State IJR Center of Aerospace Design and Additive Manufacturing, Northwestern Polytechnical University, Xi’an, China.

^6^School of Materials Science and Engineering, Northwestern Polytechnical University, Xi’an, China.

^7^International Joint Laboratory for Light Alloys (Ministry of Education), College of Materials Science and Engineering, Chongqing University, Chongqing, China.

^8^Shenyang National Laboratory for Materials Science, Chongqing University, Chongqing, China.

^9^Centre for Additive Manufacturing, School of Engineering, RMIT University, Melbourne, Victoria, Australia.

^10^Department of Materials Science and Engineering, Monash University, Clayton, Victoria, Australia.

^†^These authors contributed equally: Yingang Liu, Jingqi Zhang.

^*^Corresponding authors, e-mail: ranming.niu@sydney.edu.au (Ranming Niu), christopher.hutchinson@monash.edu (Christopher Hutchinson), [mingxing.zhang@uq.edu.au](mailto:mingxing.zhang@uq.edu.au) (Ming-Xing Zhang)

**Table of Contents**

[**Supplementary Fig. 1 | SEM micrographs and EDS analysis of LaB_6_ nanoparticles doped Cu powder feedstock.** 4](#_Toc147166526)

[**Supplementary Fig. 2 | Micro-CT characterization of the L-PBF fabricated pure Cu, 0.5LaB_6_-Cu and 1.0LaB_6_-Cu.** 5](#_Toc147166527)

[**Supplementary Fig. 3 | SEM images of top surface morphology of the L-PBF fabricated 1.0LaB_6_-Cu by using different parameters.** 6](#_Toc147166528)

[**Supplementary Fig. 4 | XRD analysis of the L-PBF fabricated pure Cu and 1.0LaB_6_-Cu.** 7](#_Toc147166529)

[**Supplementary Fig. 5 | Atom probe tomography (APT) characterization of the L-PBF fabricated 1.0LaB_6_-Cu.** 8](#_Toc147166530)

[**Supplementary Fig. 6 | TEM characterization of** **the L-PBF fabricated 1.0LaB_6_-Cu.** 9](#_Toc147166531)

[**Supplementary Fig. 7 | Characterization of 1.0 wt% LaB_6_ microparticles and 0.5-2.0 wt% LaB_6_ nanoparticles doped Cu parts.** 10](#_Toc147166532)

[**Supplementary Fig. 8 | Nanoparticles in the top surface of the L-PBF fabricated 1.0LaB_6_-Cu.** 11](#_Toc147166533)

[**Supplementary Fig. 9 | Softening resistance of the L-PBF fabricated 1.0LaB_6_-Cu.** 12](#_Toc147166534)

[**Supplementary Fig. 10 | EBSD analysis of the L-PBF fabricated 1.0LaB_6_-Cu before and after annealing.** 13](#_Toc147166535)

[**Supplementary Fig. 11 | Comparison of tensile properties of AM fabricated pure Cu using green laser and electron beam with that of 1.0LaB_6_-Cu.** 22](#_Toc147166544)

[**Supplementary Fig. 12 | SEM image of LaB_6_ nanoparticles after annealing at 1,050 ^o^C.** 23](#_Toc147166546)

[**Supplementary Fig. 13 | Characterization of TiB_2_ nanoparticles doped Cu powder feedstock and the L-PBF fabricated part.** 24](#_Toc147166548)

[**Supplementary Table 1 | L-PBF processing parameters.** 14](#_Toc147166536)

[**Supplementary Table 2 | Measured chemical compositions (in wt%) of the L-PBF fabricated pure Cu and 1.0LaB_6_-Cu.** 15](#_Toc147166537)

[**Supplementary Table 3 | Physical properties of commonly used ceramics.** 16](#_Toc147166538)

[**Supplementary Note 1 − Selection of additive.** 17](#_Toc147166539)

[**Supplementary Note 2 − Uniform dispersion of re-precipitated LaB_6_ nanoparticles.** 17](#_Toc147166540)

[**Supplementary Note 3 − Characterization using APT.** 19](#_Toc147166541)

[**Supplementary Note 4 − Strengthening mechanisms of the L-PBF fabricated 1.0LaB_6_-Cu.** 20](#_Toc147166542)

[**Supplementary Note 5 – Comparison with AM fabricated pure Cu using green laser and electron beam.** 21](#_Toc147166543)

[**Supplementary Note 6 – Variation of strength and ductility of the 1.0LaB_6_-Cu after annealing.** 22](#_Toc147166545)

[**Supplementary Note 7 − Parallel experiment of the L-PBF fabricated 1.0TiB_2_-Cu.** 23](#_Toc147166547)

[**Supplementary References** 24](#_Toc147166549)


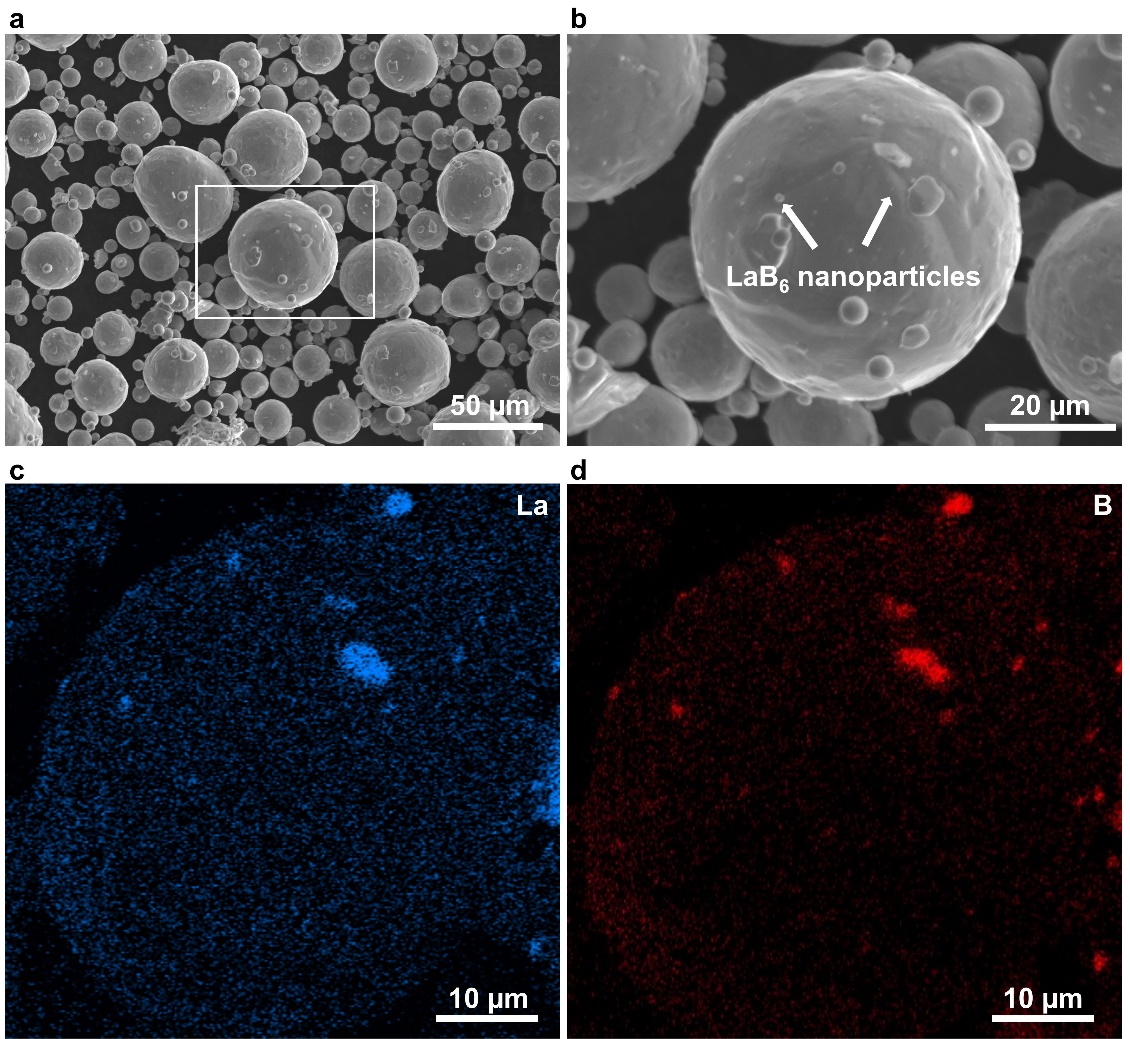


## **Supplementary Fig. 1 | SEM micrographs and EDS analysis of LaB_6_ nanoparticles doped Cu powder feedstock. a**, 1.0 wt% LaB_6_ nanoparticles doped Cu powder feedstock. **b**, High magnification SEM image corresponding to the white frame in (**a**). **c**, **d**, EDS elemental mapping of pure Cu with addition of 1.0 wt% LaB_6_ nanoparticles. Overall uniform distribution of LaB_6_ nanoparticles with irregular shapes on the surface of Cu particles is observed after mechanical mixing.


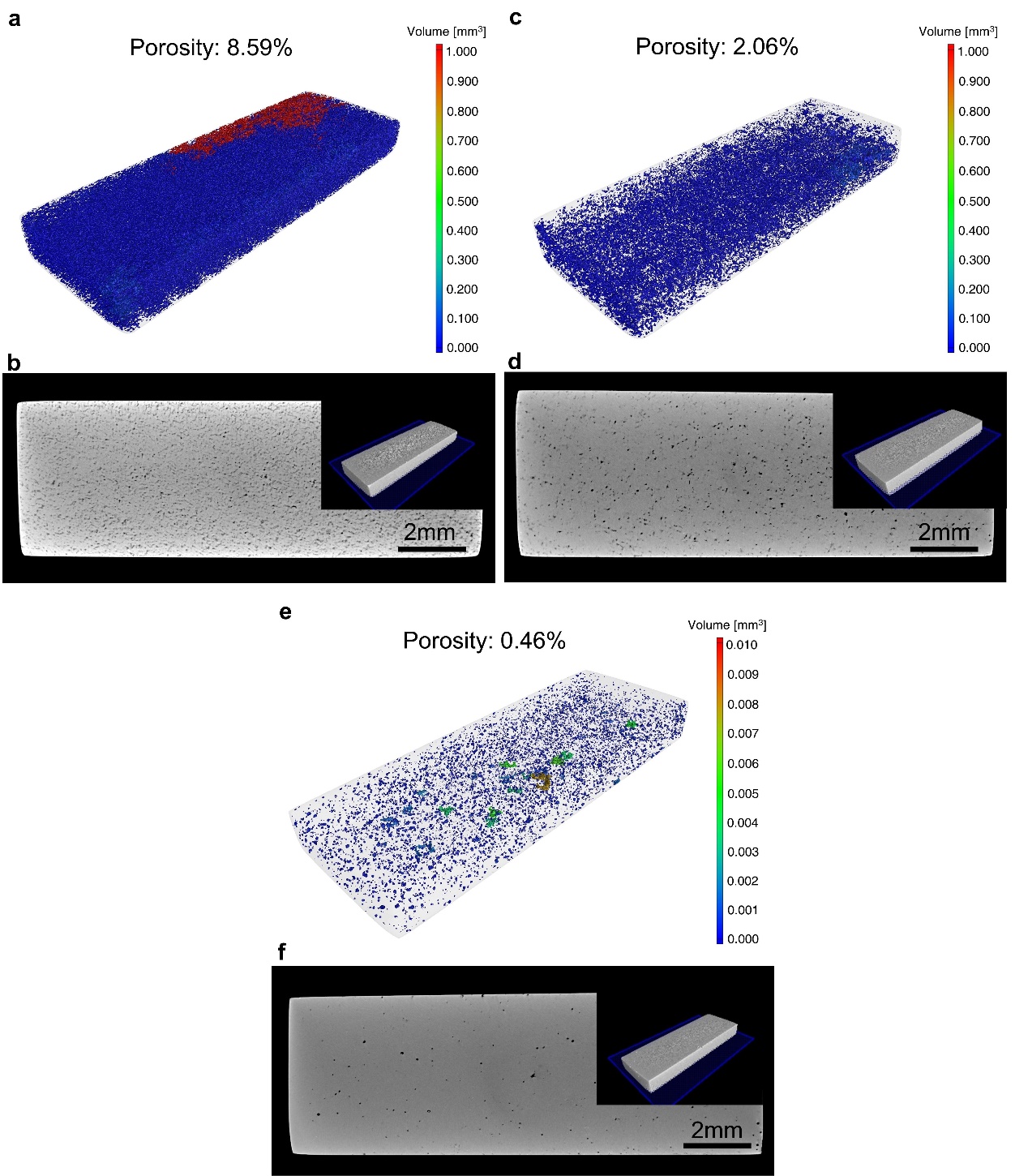


## **Supplementary Fig. 2 | Micro-CT characterization of the L-PBF fabricated pure Cu, 0.5LaB_6_-Cu and 1.0LaB_6_-Cu. a**, **b**, Micro-CT three-dimensional (3D) image (**a**) and two-dimensional (2D) slice (**b**) showing the lack-of-fusion defects in the L-PBF fabricated pure Cu. **c**, **d**, Micro-CT 3D image (**c**) and 2D slice (**d**) showing the lack-of-fusion defects in the L-PBF fabricated 0.5LaB_6_-Cu. **e**, **f**, Micro-CT 3D image (**e**) and 2D slice (**f**) showing the pores in the L-PBF fabricated 1.0LaB_6_-Cu. The measured overall porosity of parts significantly reduces from 8.59% for the L-PBF fabricated pure Cu to 0.46% for the 1.0LaB_6_-Cu.


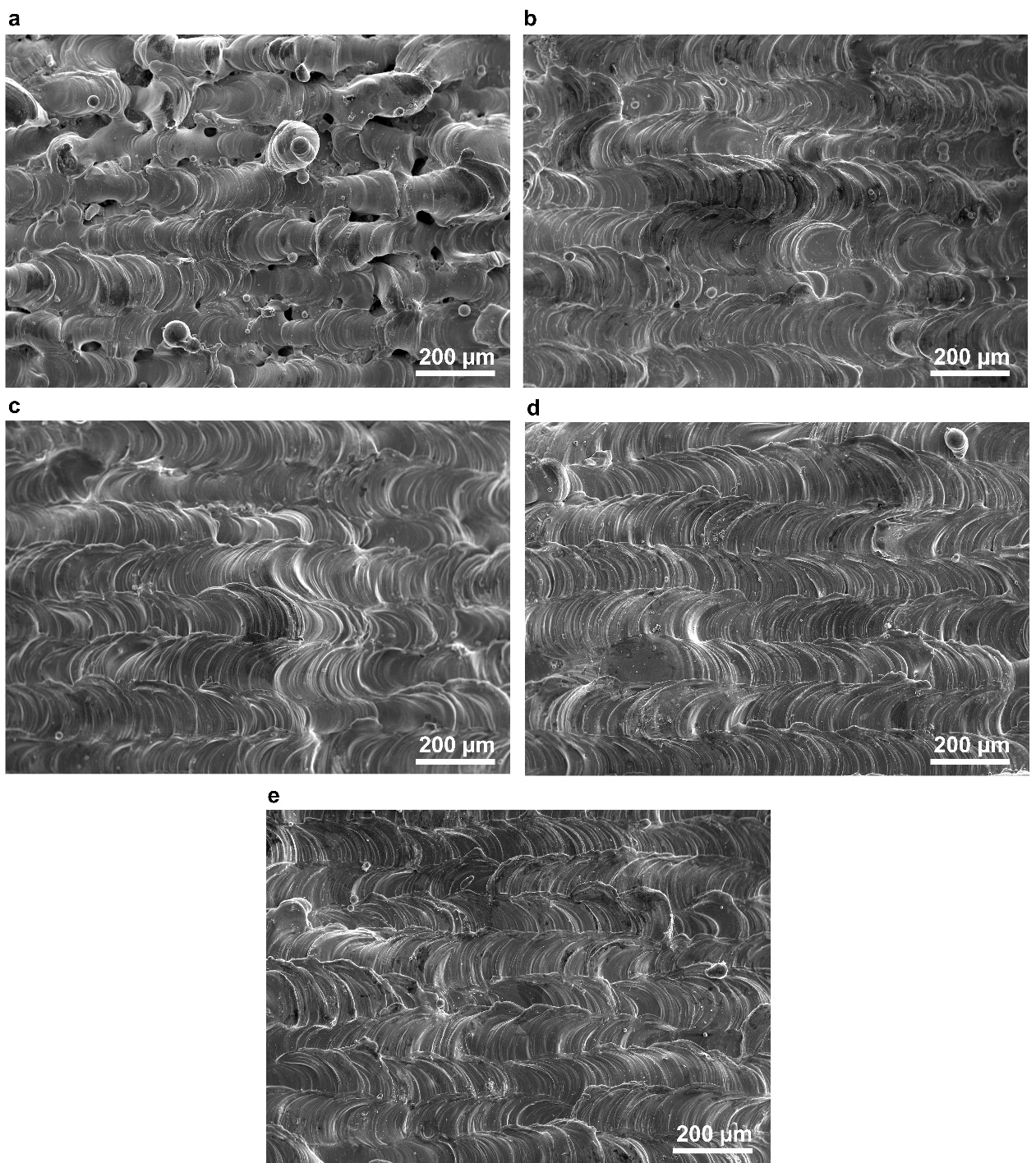


## **Supplementary Fig. 3 | SEM images of top surface morphology of the L-PBF fabricated 1.0LaB_6_-Cu by using different parameters. a**, Laser power of 300 W, scanning speed of 400 mm s^-1^, hatch spacing of 120 μm, and layer thickness of 30 μm. **b**, Laser power of 325 W, scanning speed of 400 mm s^-1^, hatch spacing of 120 μm, and layer thickness of 30 μm. **c**, Laser power of 350 W, scanning speed of 400 mm s^-1^, hatch spacing of 120 μm, and layer thickness of 30 μm. **d**, Laser power of 400 W, scanning speed of 400 mm s^-1^, hatch spacing of 120 μm, and layer thickness of 30 μm. **e**, Laser power of 375 W, scanning speed of 400 mm s^-1^, hatch spacing of 100 μm, and layer thickness of 30 μm. At a scanning speed of 400 mm s^-1^ and layer thickness of 30 μm, the well-defined laser scanning tracks are observed on the top surface of 1.0LaB_6_-Cu at a laser power above 325 W.


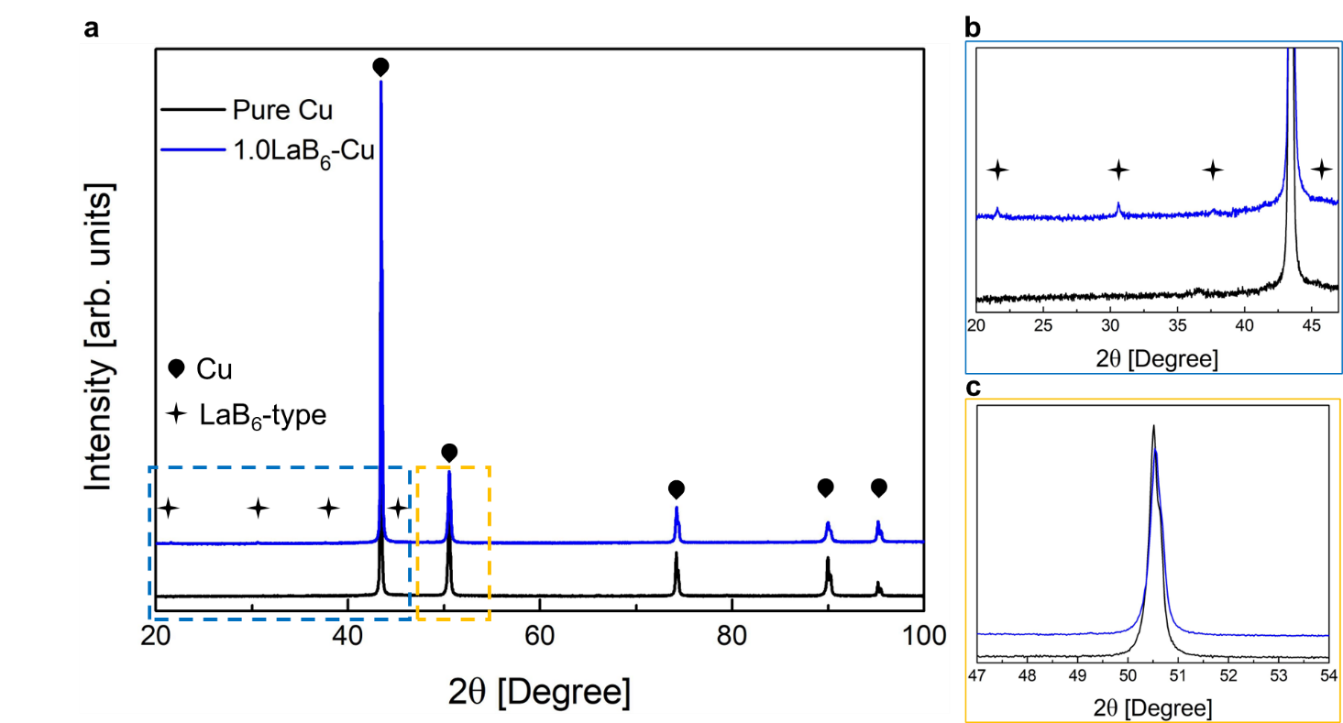


## **Supplementary Fig. 4 | XRD analysis of the L-PBF fabricated pure Cu and 1.0LaB_6_-Cu. a**, XRD spectra of pure Cu and 1.0LaB_6_-Cu. **b**, Enlarged image corresponding to the blue dotted area in (**a**). Only the diffraction peaks corresponding to Cu and LaB_6_ are detected. **c**, Enlarged image corresponding to the yellow dotted area in (**a**). XRD peak shift is not detected in 1.0LaB_6_-Cu, indicating that the lattice parameter of Cu does not change with the addition of LaB_6_. Source data are provided as a Source Data file.


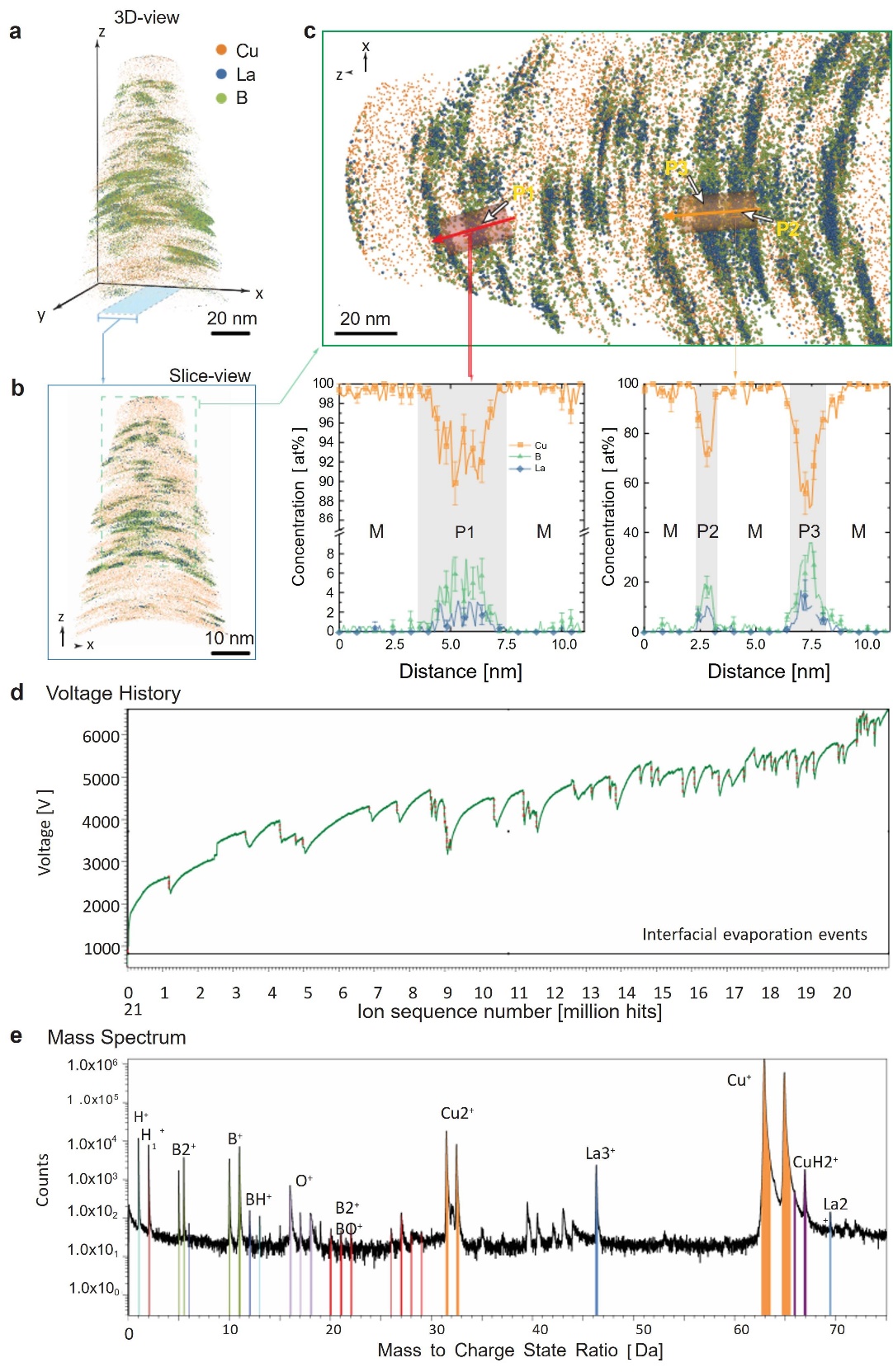


## **Supplementary Fig. 5 | Atom probe tomography (APT) characterization of the L-PBF fabricated 1.0LaB_6_-Cu. a,** 3D reconstruction of Cu, La and B distribution in the L-PBF fabricated 1.0LaB_6_-Cu. **b**, Vertical slice of the reconstructed APT volume. **c**, Enlarged image corresponding to the dotted frame in (**b**) and proximity histograms across P1, P2 and P3 showing depletion of La and B in Cu matrix. The error bars denote the standard deviation of the mean. **d,** Voltage histroy of dataset. This image showing significnat voltage fluctation caused by the different evaportaion field of LaB_6_ and Cu. **e,** APT mass spectrum and indexing used for data analysis, data binned in width of 0.015 Da for display. M: Matrix, P: Precipitate. Source data are provided as a Source Data file.


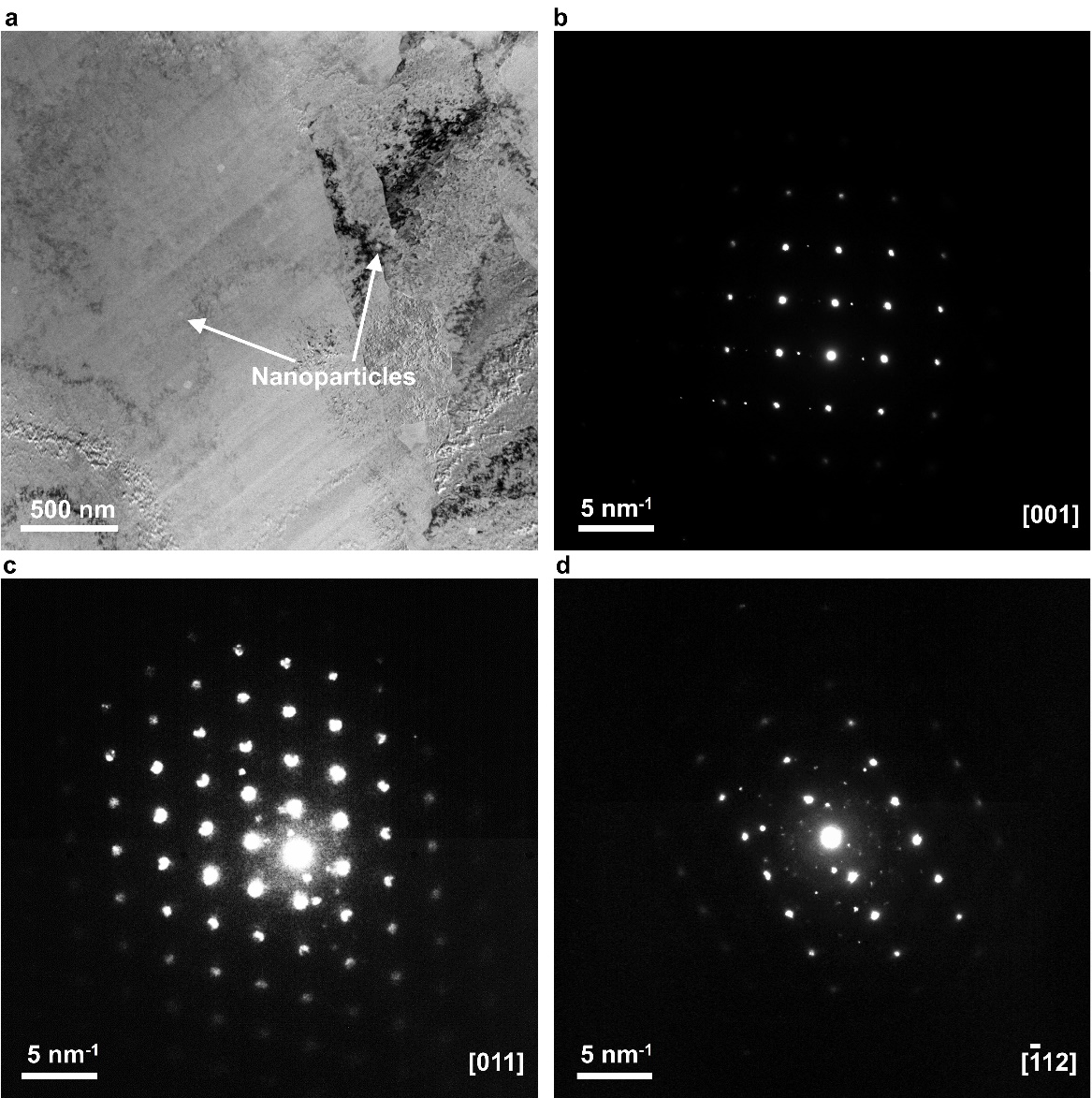


## **Supplementary Fig. 6 | TEM characterization of** **the L-PBF fabricated 1.0LaB_6_-Cu. a**, Bright-field (BF) TEM image showing the LaB_6_ nanoparticles. **b**,**c**,**d**, Selected area electron diffraction (SAED) patterns corresponding to (**a**) obtained from different zone axes. It is evident that there is no specific orientation relationship between LaB_6_ nanoparticles and Cu matrix.


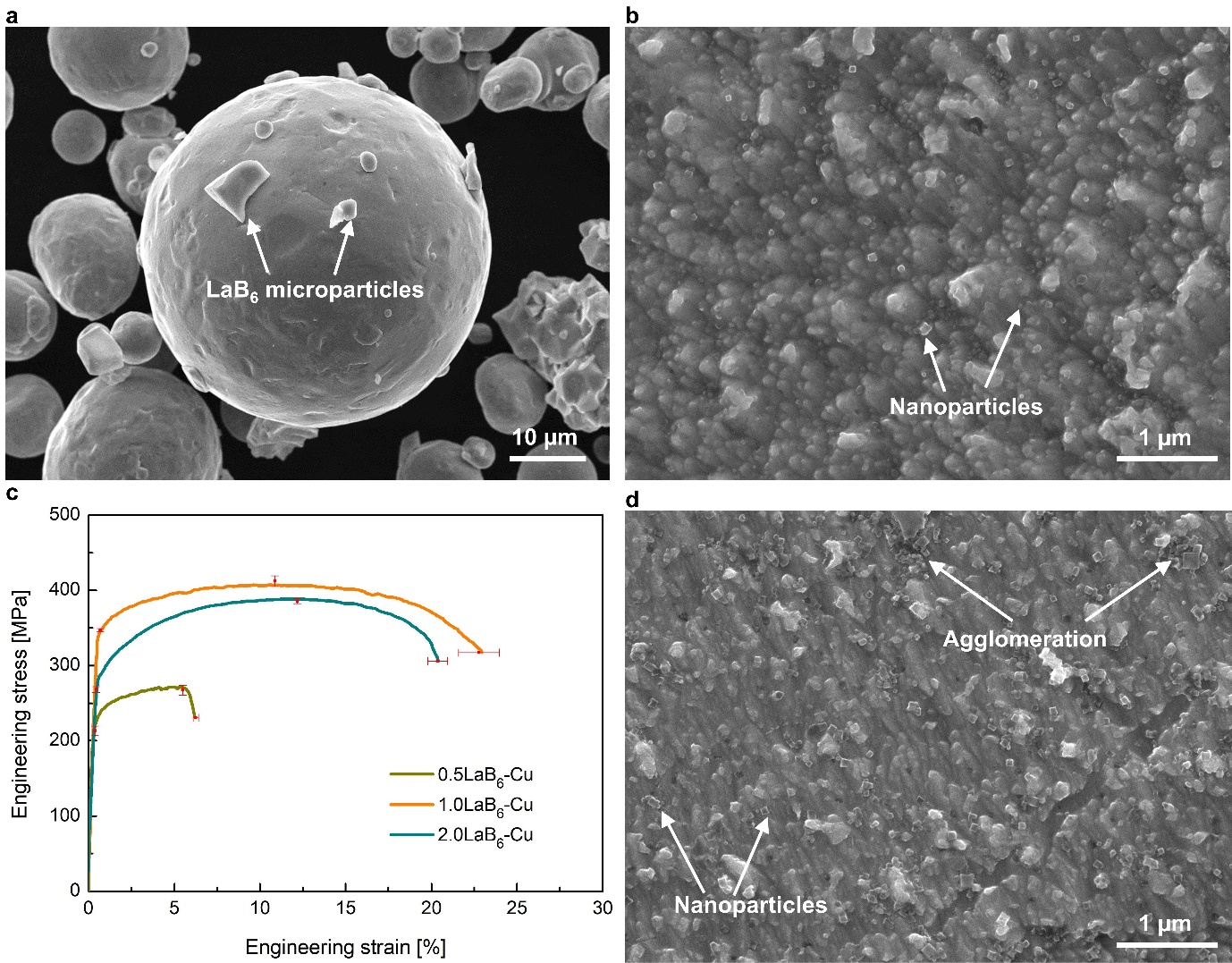


## **Supplementary Fig. 7 | Characterization of 1.0 wt% LaB_6_ microparticles and 0.5-2.0 wt% LaB_6_ nanoparticles doped Cu parts. a**, Pure Cu powder with 1.0 wt% LaB_6_ microparticles (˂10 μm). After mechanical mixing, LaB_6_ microparticles homogeneously adhered to the surface of Cu particles. **b**, SEM image showing the distribution of nanoparticles in the L-PBF fabricated LaB_6_ microparticles doped Cu. The initial microparticles with irregular shapes disappeared, but LaB_6_ nanoparticles are observed. **c**, Tensile engineering stress-strain curves of the L-PBF fabricated Cu with different additions of LaB_6_ nanoparticles. The error bars represent the standard deviation of the mean. **d**, SEM image showing the nanoparticle agglomeration in the L-PBF fabricated 2.0LaB_6_-Cu. Source data are provided as a Source Data file.


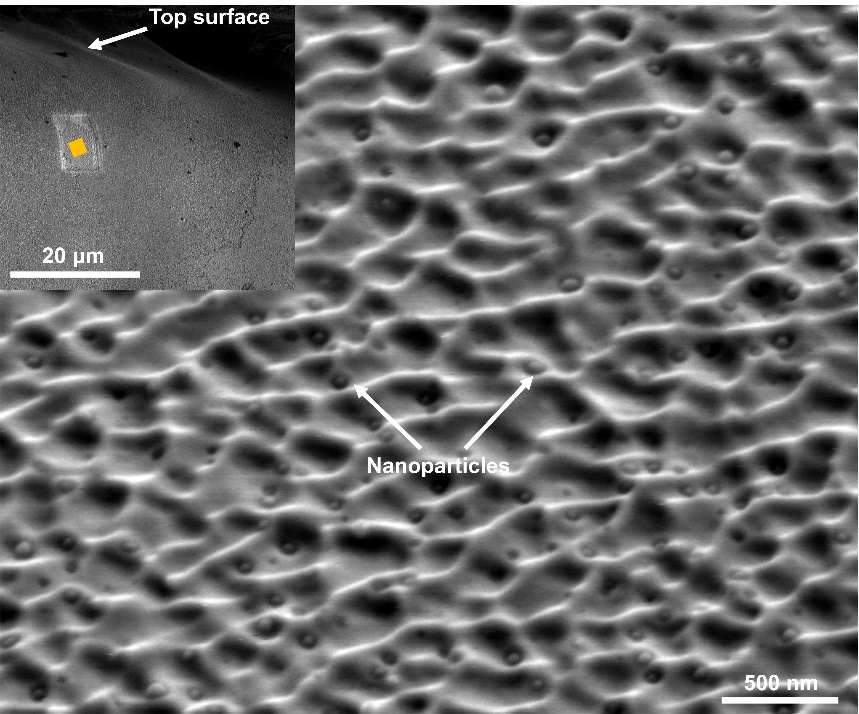


## **Supplementary Fig. 8 | Nanoparticles in the top surface of the L-PBF fabricated 1.0LaB_6_-Cu.** SEM image corresponding to the frame area in the inset showing the nanoparticles in the top surface. Large number of uniformly dispersed nanoparticles are also visible in the topmost surface layer which is free from extensive thermal cycling during L-PBF.


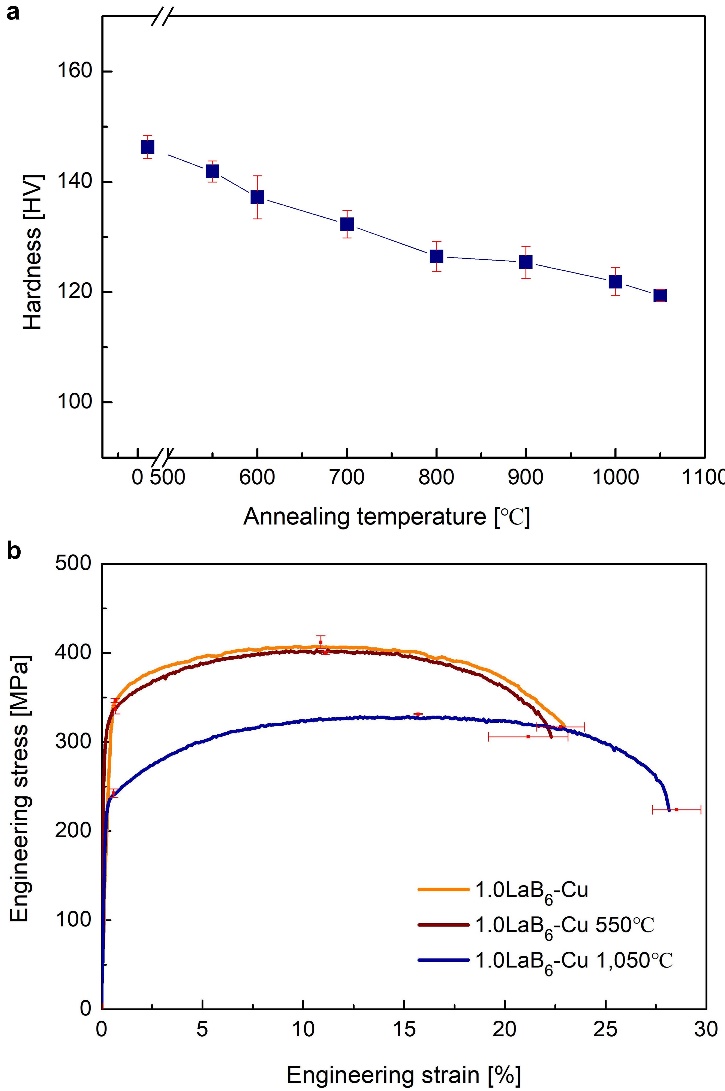


## **Supplementary Fig. 9 | Softening resistance of** **the L-PBF fabricated 1.0LaB_6_-Cu. a**, The hardness of the L-PBF fabricated 1.0LaB_6_-Cu subjected to thermal exposure at elevated temperatures. **b**, Tensile engineering stress-strain curves of the L-PBF fabricated 1.0LaB_6_-Cu subjected to thermal exposure at 550 ^o^C and 1,050 ^o^C. The error bars represent the standard deviation of the mean. Source data are provided as a Source Data file.


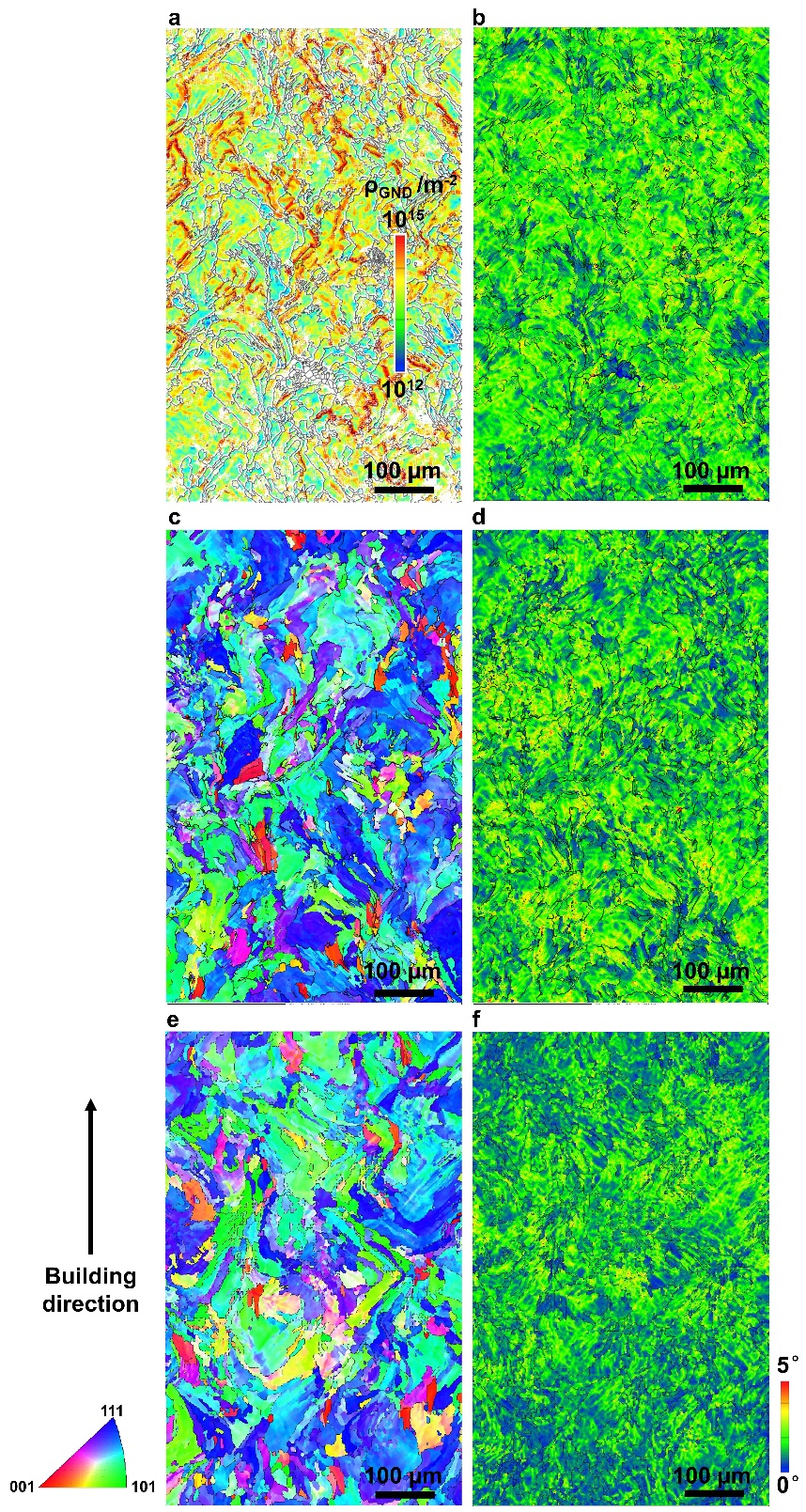


## **Supplementary Fig. 10 | EBSD analysis of the L-PBF fabricated 1.0LaB_6_-Cu before and after annealing. a**, The geometrically necessary dislocation (GND) density of the L-PBF fabricated 1.0LaB_6_-Cu calculated from Kernel average misorientation (KAM) data in (**b**). The GND density is estimated to be 8.03×10^13^ m^-2^. **b**, KAM image of the L-PBF fabricated 1.0LaB_6_-Cu corresponding to the EBSD inverse pole figure (IPF) map in Fig. 1d. **c**, EBSD IPF map of the L-PBF fabricated 1.0LaB_6_-Cu after annealing at 550 ℃ for 1 h. **d**, KAM image corresponding to (**c**). **e**, EBSD IPF map of the L-PBF fabricated 1.0LaB_6_-Cu after annealing at 1,050 ℃ for 1 h. **f**, KAM image corresponding to (**e**). Decreased dislocation density confirmed by the lower KAM value of the L-PBF fabricated 1.0LaB_6_-Cu after annealing at 1,050 ℃ leads to the reduction in the tensile strength and the increase in the ductility.

## **Supplementary Table 1 | L-PBF processing parameters.**

| L-PBF processing parameters | Value | Unit |
| --- | --- | --- |
| Laser power | 375 | W |
| Scanning speed | 400 | mm s^-1^ |
| Layer thickness | 30 | µm |
| Hatch spacing | 120 | µm |
| Spot size | 80 | µm |
| Preheating temperature | 200 | °C |
| Oxygen concentration | <0.05 | vol% |

## **Supplementary Table 2 | Measured chemical compositions (in wt%) of the L-PBF fabricated pure Cu and 1.0LaB_6_-Cu.**

| Specimen | La | B | O | Cu |
| --- | --- | --- | --- | --- |
| Pure Cu | / | / | 0.048 | Bal. |
| 1.0LaB_6_-Cu | 0.73 | 0.30 | 0.014 | Bal. |

## **Supplementary Table 3 | Physical properties of commonly used ceramics**^1-6^**.**

| Ceramic | Wetting angle of liquid Cu on ceramics [°] | Melting temperature [°C] |
| --- | --- | --- |
| Al_2_O_3_^1^ | 128 | 2072 |
| SiO_2_^1^ | 128 | 1710 |
| MgO^1^ | 133 | 2852 |
| Y_2_O_3_^1^ | 140 | 2425 |
| TiC^1,2^ | 112 | 3100 |
| ZrC^1,2^ | 127 | 3532 |
| HfC^1,2^ | 134 | 3900 |
| VC^1,2^ | 50 | 2810 |
| NbC^1,2^ | 70 | 3490 |
| TaC^1,2^ | 78 | 3880 |
| WC^2,3^ | 30 | 2870 |
| B_4_C^2,3^ | 136 | 2445 |
| TiN^2,3^ | 155 | 2950 |
| ZrN^2,3^ | 148 | 2980 |
| HfN^2,3^ | / | 3387 |
| NbN^2,3^ | 150 | 2573 |
| TaN^2,3^ | / | 3093 |
| TiB_2_^1,4^ | 143 | 3230 |
| ZrB_2_^1,4^ | 142 | 3000 |
| NbB_2_^2,3^ | 131 | 3050 |
| TaB_2_^1,4^ | 77 | 3000 |
| LaB_6_^5,6^ | 71 | 2210 |

## **Supplementary Note 1 − Selection of additive.**

Within the category of commonly used ceramics, encompassing oxides, carbides, nitrides, and borides (Supplementary Table 3), LaB_6_ stands out due to its notably lower melting point of 2,210°C. LaB_6_ has previously found application as a grain refiner in additive manufacturing for aluminium alloys^7^, yet its utilization in Cu remains largely unexplored. La and B exhibit negligible solid solubility in Cu, which means any solute trapping in the solid can be minimised and detrimental effects on the conductivity can be minimised. The low melting point (compared to other possible additions) improves the potential for complete dissolution of the particles in the melt pools, followed by subsequent re-precipitation during solidification (due to the negligible solid solubility of La and B). This is the key design idea (dissolution of the LaB_6_ and complete re-precipitation) that drives this work. LaB_6_ also possesses a relatively low wetting angle of 71° when contacting with liquid Cu, affirming that it fulfils the proposed selection criteria.

## **Supplementary Note 2 − Uniform dispersion of re-precipitated LaB_6_ nanoparticles.**

Three interactions between nanoparticles are commonly considered in molten metal^8^: van der Waals potential, Brownian motion energy, and interfacial energy. Good dispersion of ceramic nanoparticles can be achieved in molten metal by synergistically reducing attractive van der Waals forces, providing high thermal energy for dispersion, and creating a high energy barrier to prevent clustering.

*Attractive van der Waals force.* For two LaB_6_ nanoparticles in a Cu melt, the van der Waals interaction can be approximately estimated by the following equation^8,9^.

$$W_{\text{vdw}}=\frac{\sqrt{A_{\text{LaB}_{6}}}-\sqrt{A_{\text{Cu}}}}{6D}\cdot\frac{R}{2}$$

where *A* is the Hamaker constant, *D* is the distance between two nanoparticles, and *R* is the radius of LaB_6_ nanoparticles (23 nm). Equation 1 holds true under the condition that two nanoparticles interact within molten Cu, where D is approximately greater than the thickness of two atomic layers (~ 0.4 nm). *A*_Cu_ is 410 zJ^10^. *A*_LaB6_ is probably in the range from 200 to 500 zJ^9^. *W*_vdw_ will therefore range from -58. 5 to 0 zJ.

*Thermal energy for dispersion.* Thermal energy makes nanoparticles disperse by Brownian motion and can be calculated as follows^8^.

$$E_{\text{b}}=kT$$

where *k* is the Boltzmann constant (1.380649×10^-23^ J K^-1^); *T* is the absolute temperature. As LaB_6_ can be melted during printing, ~2,500 K can be achieved in the melt pool of the L-PBF fabricated 1.0LaB_6_-Cu, and *E*_b_ is estimated to be 34.5 zJ.

*Energy barrier preventing clustering.*  Good wetting between molten Cu and LaB_6_ nanoparticles creates an energy barrier to reduce the possibility of nanoparticles being in direct contact with each other. The energy barrier can be calculated using the following equation^8,9^:

$$W_{\text{barrier}}=S(\sigma_{\text{LaB}_{6}}-\sigma_{\text{LaB}_{6}\text{-Cu}})=S\sigma_{\text{Cu}}\cdot\cos\theta$$

where *S* is the effective area, *σ*_LaB6_ is the surface energy of the LaB_6_, *σ*_LaB6-Cu_ is the interfacial energy between the ceramic particles and molten Cu, *σ*_Cu_ is the surface tension of molten Cu (1.3 J m^-2^)^11^, and *θ* is the wetting angle of molten Cu on LaB_6_ surface. This equation clearly suggests that the better the wetting of the molten Cu on ceramics nanoparticles (smaller *θ*), the higher the energy barrier to prevent clustering of ceramic nanoparticles. According to the Langbein approximation^8,12^, the effective interaction area of two spheres is given by

$$S=\pi RD_{0}$$

where *D*_0_ = 0.2 nm. As LaB_6_ exhibits a relatively low wetting angle of 71° with molten Cu (Supplementary Table 3), *W*_barrier_ is estimated to be 6,116.4 zJ. The calculation suggests that *W*_barrier_ would be much higher than *W*_vdw_, which will favour a uniform dispersion of re-precipitated LaB_6_ nanoparticles in Cu melt during L-PBF of 1.0LaB_6_-Cu.

Although nanoparticles can be uniformly dispersed in solid Cu via precipitation in the solidified Cu, this requires supersaturation of the constituent elements, which degrades the conductivity of Cu^13-15^. Furthermore, post-AM heat treatment may be required to promote the precipitation, restore the electrical conductivity, and strengthen the Cu^13-15^, leading to longer production cycles. In addition, previous work^16,17^ indicated that the externally added nano- or micro- particles tend to segregate along the melt pool boundaries if they do not dissolve into the melt even though there is strong convection in the melt pool. Hence, re-precipitation during solidification is designed to achieve both high strength and high conductivity without the need for post-AM heat treatment, distinguishing it from solid-state phase transformation.

## **Supplementary Note 3 − Characterization using APT.**

As presented in Fig. 3, the APT determined maximum apparent ratio of B to La in P1 and P2 are 3.9:1 and 2.8:1, respectively, which are lower than the stoichiometric ratio of 6:1 (see also the extra dataset in Supplementary Fig. 5). Such detecting deficiency of B using APT has also been reported in single crystalline LaB_6_ by Murakami et al.^18^, showing a significant reduction in apparent B composition in APT data because of the disturbing hydrogen. It has been recognised that the formation of boron hydride reduces the evaporation field of B, and consequently lowers the detecting efficiency^18^. As hydrogen presents in most APT chambers due to its high diffusivity^19,20^, the quantification of B is thereby affected, as demonstrated by clear BH^2+^ peaks at 12 and 13 Da on the mass spectrum (Supplementary Fig. 5e). In addition, it appears that the evaporation fields for the nanoparticles and Cu matrix are significantly different, as confirmed by the extremely serrated voltage curve (see Supplementary Fig. 5d). The bumpy evaporation field causes significant trajectory aberration, which makes the quantification of B even more difficult. Nevertheless, APT data approximately shows that La and B atoms preferentially form nanoparticles rather than dissolving in the Cu matrix.

*Evaluation of the sensitivity of the APT test.*  The mass resolution^21,22^, *m*/Δ*m*, is one of the most important metrics by which APT performance is measured, where *m* is the mass-to-charge state ratio for the peak and Δ*m* is the width of the peak measured at a percentage the peak maximum, e.g. full-width at half-maximum (FWHM), full-width at tenth-maximum (FW0.1M). The FWHM mass resolution for the major ^11^B^+^ peak at 11 Da is 200 and 330 for the dataset presented in Fig. 3 and Supplementary Fig. 5, respectively. Note that the mass resolution can be affected by a variety of analysis conditions of the sample, for example, the pulse fraction, the temperature, the pulsing mode, and the geometry of the tip^21,22^. APT parameters given in Methods are to the best compensate the data yielding rate of the experiment and the mass resolution.

## **Supplementary Note 4 − Strengthening mechanisms of the L-PBF fabricated 1.0LaB_6_-Cu.**

In comparison to the annealed pure Cu (yield strength of 80 MPa^23^), the L-PBF fabricated 1.0LaB_6_-Cu was significantly strengthened (yield strength of 347 MPa) (Fig. 4a). The strength enhancement is supposed to have originated from two strengthening mechanisms, including dispersion strengthening and dislocation strengthening. In general, the dislocation- nanoparticle interaction can be coordinated with dislocation bypass in an Orowan mode for large and hard particles and/or incoherent particles. As for the hard LaB_6_ in this work, dispersion strengthening could occur through dislocations bypass of the homogeneously dispersed LaB_6_ nanoparticles. In addition, a high density of dislocations is usually found in the L-PBF fabricated parts, which is associated with the heating-cooling cycles stemmed from L-PBF^24^. This is also true for the L-PBF fabricated 1.0LaB_6_-Cu (Supplementary Fig. 10a, b), leading to a dislocation strengthening contribution.

## **Supplementary Note 5 – Comparison with AM fabricated pure Cu using green laser and electron beam.**

In comparison to the infrared laser, AM using a green laser provides a pathway to produce highly dense Cu parts because the laser absorptivity of pure Cu can be substantially improved with a lower wavelength of about 515 nm^25,26^. However, metal 3D printers equipped with the green laser are not currently commercially available^27,28^. More importantly, pure Cu is intrinsically soft and shows significantly softening at elevated temperatures. This has been a long-standing issue as most commonly used strengthening methods reduce the conductivity of pure Cu. Although the green laser 3D printer can be used to create pure Cu parts with a high density, the metal is still soft. Alternatively, AM with an electron beam (EB) enables the fabrication of high-density pure Cu parts^29^. Like the green laser, pure Cu components by EB-based AM technology typically show low strength and the inability to resist thermal softening at elevated temperatures. The tensile properties of 3D printed pure Cu using the green laser and electron beam are compared to those of 1.0LaB_6_-Cu in our study as shown in Supplementary Fig. 11. Although 3D printing with a green laser or an electron beam produces pure Cu parts with high ductility and conductivity^25,26,30-32^, their strength (yield strength of 69-180 MPa) is as low as that of annealed pure Cu. By contrast, our work achieved much higher strength (yield strength of 347 MPa) without significantly reducing the conductivity (98.4% IACS).


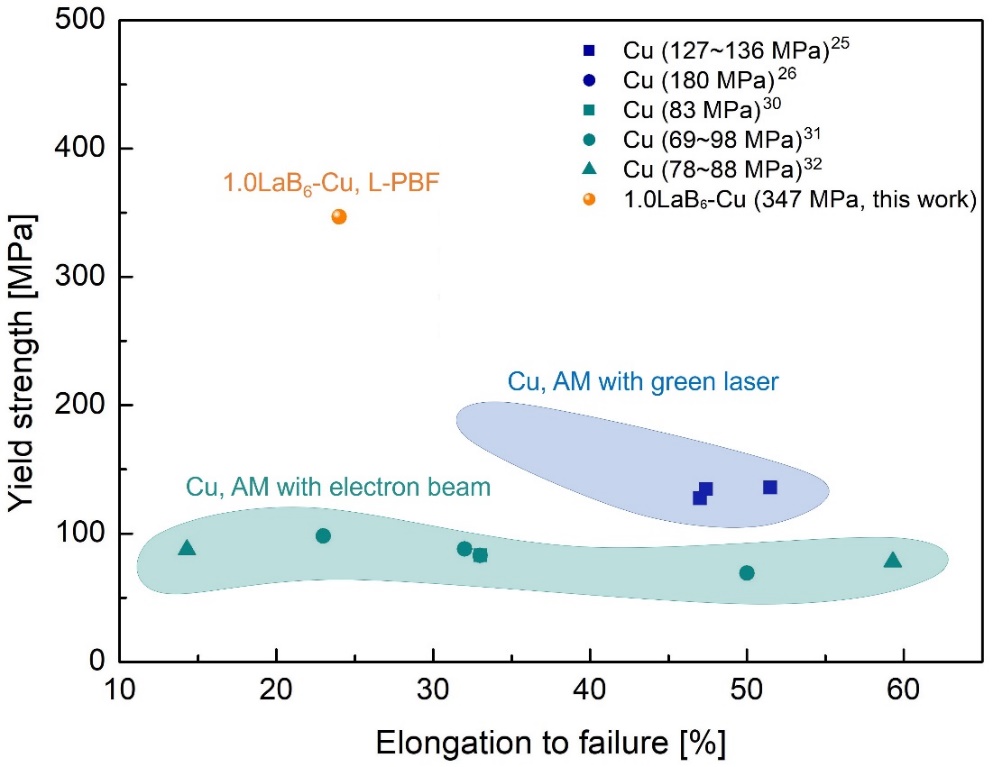


## **Supplementary Fig. 11 | Comparison of tensile properties of AM fabricated pure Cu using green laser and electron beam with those of 1.0LaB_6_-Cu.** 1.0LaB_6_-Cu exhibited significantly greater yield strength compared to pure Cu produced through AM using both green laser and electron beam.

## **Supplementary Note 6 – Variation of strength and ductility of the 1.0LaB_6_-Cu after annealing.**

Annealing at 550 ^o^C marginally changed the mechanical performance of the L-PBF fabricated 1.0LaB_6_-Cu (Supplementary Fig. 9). ~80% of the ultimate tensile strength of the L-PBF fabricated 1.0LaB_6_-Cu can be retained with further increasing the annealing temperature to 1,050 ^o^C, while a larger elongation to failure of 28% was achieved (Supplementary Fig. 9b). Compared with the L-PBF fabricated and low-temperature annealed 1.0LaB_6_-Cu, there is no obvious variation in the grain morphology and size after annealing at 1,050 ^o^C (Fig. 1a_3_, Supplementary Fig. 10). Additionally, SEM characterization demonstrates that such a high annealing temperature does not result in any obvious particle coarsening (Supplementary Fig. 12). The strength loss observed is due to a reduction in the dislocation density (static recovery), as revealed by EBSD (Supplementary Fig. 10f). Accordingly, an increase in the ductility is visible in Supplementary Fig. 9b.


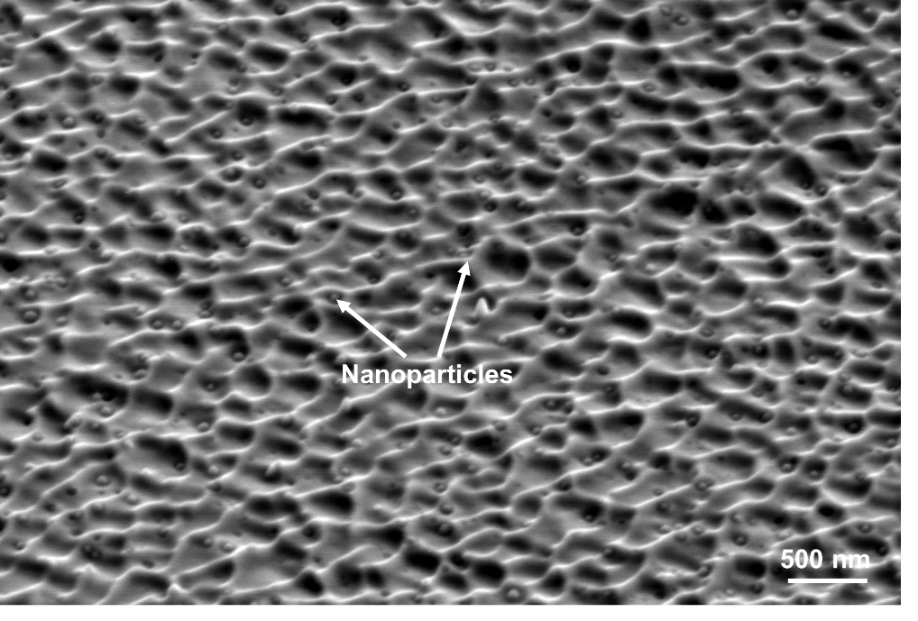


## **Supplementary Fig. 12 | SEM image of LaB_6_ nanoparticles after annealing at 1,050 ^o^C.** The LaB_6_ nanoparticles in the L-PBF fabricated 1.0LaB_6_-Cu exhibit no apparent coarsening following annealing at 1,050 ^o^C.

## **Supplementary Note 7 − Parallel experiment of the L-PBF fabricated 1.0TiB_2_-Cu.**

To further validate our design strategy, we carried out a parallel experiment using TiB_2_ nanoparticles with the same particle size and addition level (Methods). Our laser reflectivity test exhibits a prominent step around the 840 nm wavelength (Supplementary Fig. 13b). Although this has been previously reported and documented^33^, the actual causes are unclear. Because our current focus lies in the laser reflectivity at a wavelength of 1,060 nm, as used by the L-PBF system in our study, we may have to leave this phenomenon for future study. The measured laser reflectivity of pure Cu at wavelength of 1,060 nm is 78%, which closely aligns with the reported values in the range of 74%-79%^27,34,35^. The reflectivity exhibited a noticeable decrease with the introduction of 1 wt% TiB_2_ nanoparticles. Compared to LaB_6_, TiB_2_-doped Cu powder feedstock has a higher laser absorptivity (Supplementary Fig. 13b) and hence the as-fabricated 1.0TiB_2_-Cu part exhibits high density without any lack-of-fusion defects (Supplementary Fig. 13c). However, the SEM examination demonstrates that, unlike the LaB_6_, agglomeration of TiB_2_ nanoparticles took place along the grain boundaries (Supplementary Fig. 13d). This is due to the high wetting angle between the TiB_2_ and Cu melt (Supplementary Table 3). Furthermore, the relatively high melting point of TiB_2_ led to incomplete melting of the particles. The solid solubility of Ti in Cu inhibits the re-precipitation of TiB_2_ from the melt and reduce the conductivity. Therefore, the 1.0TiB_2_-Cu only achieves an ultimate tensile strength of 252 ± 4 MPa (Supplementary Fig. 13e) and an electrical conductivity of 91.2% IACS, which are much lower than the 1.0LaB_6_-Cu part. This confirms that all the selection criteria of additives are essential to produce high performance Cu parts. Given that only the commonly used ceramics were considered in this work, it is expected that other intermetallic compounds and/or other ceramics can be sought to meet the selection criteria for a range of different metals.


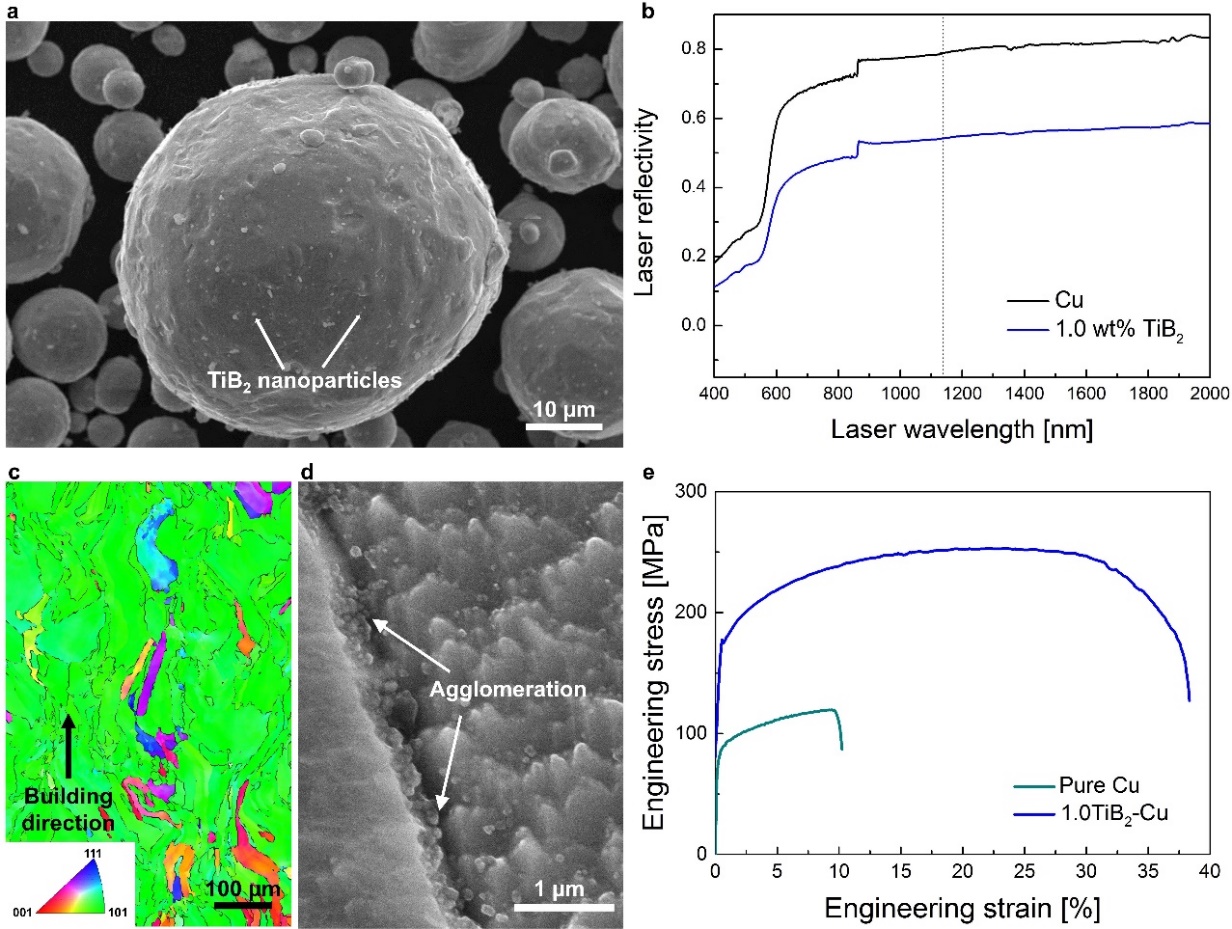


## **Supplementary Fig. 13 | Characterization of TiB_2_ nanoparticles doped Cu powder feedstock and the L-PBF fabricated part. a**, SEM image of 1.0 wt% TiB_2_ nanoparticles doped Cu powder feedstock. **b**, Laser reflectivity of pure Cu and 1.0 wt% TiB_2_ nanoparticles doped Cu powder feedstock at different laser wavelengths. After introducing 1.0 wt% TiB_2_ nanoparticles, the powder mixture shows much lower laser reflectivity than pure Cu powder. **c**, EBSD inverse pole figure (IPF) map of the L-PBF fabricated 1.0TiB_2_-Cu showing its high density. **d**, High magnification SEM image exhibiting the agglomeration of TiB_2_ nanoparticles along the grain boundary. **e**, Tensile engineering stress-strain curves of the L-PBF fabricated pure Cu and 1.0TiB_2_-Cu. Source data are provided as a Source Data file.

## **Supplementary References**

1. Eustathopoulos, N., Nicholas, M. G. & Drevet, B. *Wettability at High Temperatures*. (Elsevier, Oxford, 1999).
2. Pierson, H. O. *Handbook of Refractory Carbides and Nitrides: Properties, Characteristics, Processing and Applications*. (Noyes Publication, New York, 1997).
3. Kennedy, A. R., Wood, J. D. & Weager, B. M. The wetting and spontaneous infiltration of ceramics by molten copper. *J. Mater. Sci.* **35**, 2909-2912 (2000).
4. Golla, B. R., Mukhopadhyay, A., Basu, B. & Thimmappa, S. K. Review on ultra-high temperature boride ceramics. *Prog. Mater. Sci.* **111**, 100651 (2020).
5. Zhou, S. *et al.* Synthesis and properties of nanostructured dense LaB_6_ cathodes by arc plasma and reactive spark plasma sintering. *Acta Mater.* **58**, 4978-4985 (2010).
6. Verkhovodov, P. A., Kuz'mina, T. I., Levchenko, G. V., Luban, R. B. & Yupko, V. L. Possibility of formulating materials based on copper and lanthanum hexaboride. *Sov. Powder Metall. Met. Ceram.* **18**, 559-562 (1979).
7. Tan, Q. *et al.* Demonstrating the roles of solute and nucleant in grain refinement of additively manufactured aluminium alloys. *Addit. Manuf.* **49**, 102516 (2022).
8. Chen, L. Y. *et al.* Processing and properties of magnesium containing a dense uniform dispersion of nanoparticles. *Nature* **528**, 539-543 (2015).
9. Cao, C. *et al.* Bulk ultrafine grained/nanocrystalline metals via slow cooling. *Sci. Adv.* **5**, eaaw2398 (2019).
10. Xu, J. Q., Chen, L. Y., Choi, H. & Li, X. C. Theoretical study and pathways for nanoparticle capture during solidification of metal melt. *J. Phys.: Condens. Matter* **24**, 255304 (2012).
11. Mills, K. C. & Su, Y. C. Review of surface tension data for metallic elements and alloys: Part 1 – Pure metals. *Int. Mater. Rev.* **51**, 329-351 (2006).
12. Israelachvili, J. N. *Intermolecular and Surface Forces*. (Academic press, California, 2011).
13. Wang, J., Zhou, X. & Li, J. Evolution of microstructures and properties of SLM-manufactured Cu-15Ni-8Sn alloy during heat treatment. *Addit. Manuf.* **37**, 101599 (2020).
14. Guan, P. *et al.* Effect of selective laser melting process parameters and aging heat treatment on properties of CuCrZr alloy. *Mater. Res. Express.* **6**, 1165 (2019).
15. Zhang, S. *et al.* Microstructure and properties in QCr0.8 alloy produced by selective laser melting with different heat treatment. *J. Alloys Compd.* **800**, 286-293 (2019).
16. Lu, J. L. et al. Compression behaviour of quasicrystal/Al composite with powder mixture driven layered microstructure prepared by selective laser melting. *Opt. Laser. Technol*. **129**, 106277 (2020).
17. Zhai, W., Zhu, Z., Zhou, W., Nai, S. M. L., & Wei, J. Selective laser melting of dispersed TiC particles strengthened 316L stainless steel. *Compos. B: Eng.* **199**, 108291 (2020).
18. Murakami, K., Adachi, T., Kuroda, T. & Nakamura, S. An atom-probe analysis of the LaB_6_(001) plane: II. Effect of hydrogen gas atmosphere. *Surf. Sci.* **176**, 327-335 (1986).
19. Redhead, P. A. Hydrogen in vacuum systems: An overview. *AIP Conf. Proc.* **671**, 243-254 (2003).
20. Chen, Y.-S. *et al.* Atom probe tomography for the observation of hydrogen in materials: A review. *Microsc. Microanal.* **29**, 1-15 (2023).
21. Gault, B., Moody, M. P., Cairney, J. M. & Ringer, S. P. *Atom Probe Microscopy*. (Springer Science & Business Media, New York, 2012).
22. Larson, D. J., Prosa, T. J., Ulfig, R. M., Geiser, B. P. & Kelly, T. F. *Local Electrode Atom Probe Tomography*. (Springer Science, New York, 2013).
23. Lu, L., Shen, Y., Chen, X., Qian, L. & Lu, K. Ultrahigh strength and high electrical conductivity in copper. *Science* **304**, 422-426 (2004).
24. Li, Z. *et al.* Enhanced strengthening and hardening via self-stabilized dislocation network in additively manufactured metals. *Mater. Today* **50**, 79-88 (2021).
25. Gruber, S., Stepien, L., López, E., Brueckner, F. & Leyens, C. Physical and geometrical properties of additively manufactured pure copper samples using a green laser source. *Materials* **14**, 3642 (2021).
26. Kang, S. -G. *et al.* Green laser powder bed fusion based fabrication and rate-dependent mechanical properties of copper lattices. *arXiv preprint arXiv:2210.13087*, (2022).
27. Jadhav, S. D. *et al.* Surface modified copper alloy powder for reliable laser-based additive manufacturing. *Addit. Manuf.* **35**, 101418 (2020).
28. Lin, Z., Dadbakhsh, S. & Rashid, A. Developing processing windows for powder pre-heating in electron beam melting. *J. Manuf. Process.* **83**, 180-191 (2022).
29. Horn, M. *et al.* Powder Bed Fusion of highly filigree copper features using a green laser. *Procedia CIRP* **111**, 81-86 (2022).
30. Dadbakhsh, S. et al. Process and geometrical integrity optimization of electron beam melting for copper. *CIRP Ann. Manuf. Technol.* **71**, 201-204 (2022).
31. Chinnappan, P. K. & Shanmugam, V. *Additive Manufacturing of Pure Copper Using Electron Beam Melting (EBM)*. (KTH Royal Institute of Technology, Stockholm, 2022).
32. Guschlbauer, R., Momeni, S., Osmanlic, F. & Körner, C. Process development of 99.95% pure copper processed via selective electron beam melting and its mechanical and physical properties. *Mater. Charact.* **143**, 163-170 (2018).
33. Difficulties in 3D printing of copper alloys and factors influencing laser absorption rates. https://www.mrj-lasermark.com/info/difficulties-in-3d-printing-of-copper-alloys-a-72961830.html, (2022).
34. Jadhav, S. D., Goossens, L. R., Kinds, Y., Van Hooreweder, B., & Vanmeensel, K. Laser-based powder bed fusion additive manufacturing of pure copper. *Addit. Manuf.* **42**, 101990 (2021).
35. Silbernagel, C. *et al.* Electrical resistivity of pure copper processed by medium-powered laser powder bed fusion additive manufacturing for use in electromagnetic applications. *Addit. Manuf.* **29**, 100831 (2019).
